# New products

**DOI:** 10.1155/S1463924696000041

**Published:** 1996


Journal of Automatic Chemistry, Vol. 18, No. 1 (January-February 1996), pp. 23-27

### New products

### Trade approval for safe weight indicator

The Salter Weigh-Tronix WI-150 Weight Indicator has achieved EU trade approval, which means it can be used in applications where goods which are being sold by weight are weighed in hazardous areas. The WI-150 is an ultra low power weight indicator, designed to meet the demand for an intrinsically safe weight indicator which can be safely installed in hazardous areas. The WI-150 is approved for use in hazardous environments by Zenlec. It has SIRA test and certification to standards BS 5501 Part 7 (EN 50020) and Part 9 (EN 50039), SCS Number EX 93C2033. The WI-150 is suitable for the harshest environments and is widely used in areas where flammable substances are being handled, such as the petro-chemical, paint production, fertilizer and chemical markets.

It is compatible with all Salter platforms and can be connected to a platform for a specified hazardous application, creating a complete, intrinsically safe weighing machine. The WI-150 is available in mains or battery powered versions. It is also fully expandable and can be interfaced with computers, printers and control equipment. As such peripheral equipment often needs to be located in a safe area, unless it has its own hazardous area approval the WI-150 includes an optional fibre optics data interface card to enable transmission of signals, via a fibre optic cable, to receiving circuitry in a safe area.

*Details from Salter Weigh-Tronix on (fax) 0121 553 0494.*

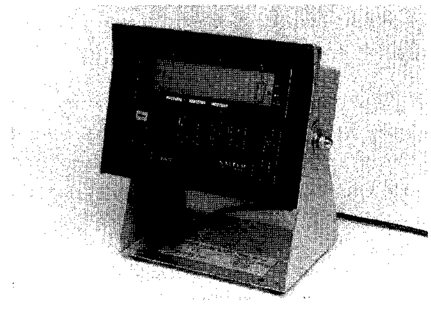

*The WI-150 which is suitable for any weighing requirements, from simple scale operations through to linking with computers, printers and data-gathering devices. It can be configured to measure in pounds, kilogrammes or litres, with a programmable density function.*

### Mass spectrometry packages for education

A range of high performance mass spectrometry packages specifically tailored for the UK academic research and teaching environment are now available from Fisons Instruments/VG Organic. With analytical instruments in higher education institutions in constant use, the instrument data system is often tied up, so it is frequently unavailable for data processing, teaching students, etc.

VG Organic's educational packages come complete with a networked data system, and incorporate a high performance notebook computer using the same Windows based MassLab 1.2 software used to run the instrument. Its flexibility as a portable system means that it can be used in the lab to access data on the hard disk of the primary data system or moved to an office or classroom to suit individual work requirements. A choice of mass spectrometry systems is available with the package:

(1) *MD 800 GC/MS:* Equipped with a proven gas chromatograph and the full range of GC/MS ionization techniques: library searchable EI ionization, molecular weight confirming positive CI and ultra-sensitive negative CI.
(2) *MD 800 LCD LC/MS:* A powerful and robust particle beam HPLC mass detector, again complete with EI, CI+ and CI− ionization sources, and which can be coupled to existing HPLC systems.
(3) *TRIO 1000 Series II LC/GC/MS:* A multiple inlet benchtop mass spectrometer with the adaptability to perform GC/MS, LC/MS or solids/DCI probe introduction. It is supplied with GC 8000, EI/CI+/CI− and *a particle beam or APcI LC/MS interface*, and can be upgraded to use other LC ionization techniques at a later date.

*For more information contact Fisons Instruments, Response Handling (IAS), Queens Avenue, Macclesfield, Cheshire SK10 2BN, UK. Tel.: 01625 434343; fax: 01625 434335.*

### 'Best instrument supplier'

Thermo Jarrell Ash, which is a subsidiary of Thermo Instrument Systems, Inc., was recently recognized by *Analytical Consumer Magazine* as one of the top ten 'best instrument suppliers' for Inductively Coupled Plasma (ICP) Spectrometers from 1990 through 1995. The award was based on interviews with instrument operators over the past five years. The satisfaction scores for Thermo Jarrell Ash's ICP spectrometers ranked significantly higher than the average of several surveys.

*For information on Thermo Jarrell Ash's analytical instruments contact Thermo Jarrell Ash, 27 Forge Parkway, Franklin, MA 02038, USA.*

0142-0453/96 $12.00 © 1996 Taylor & Francis Ltd.

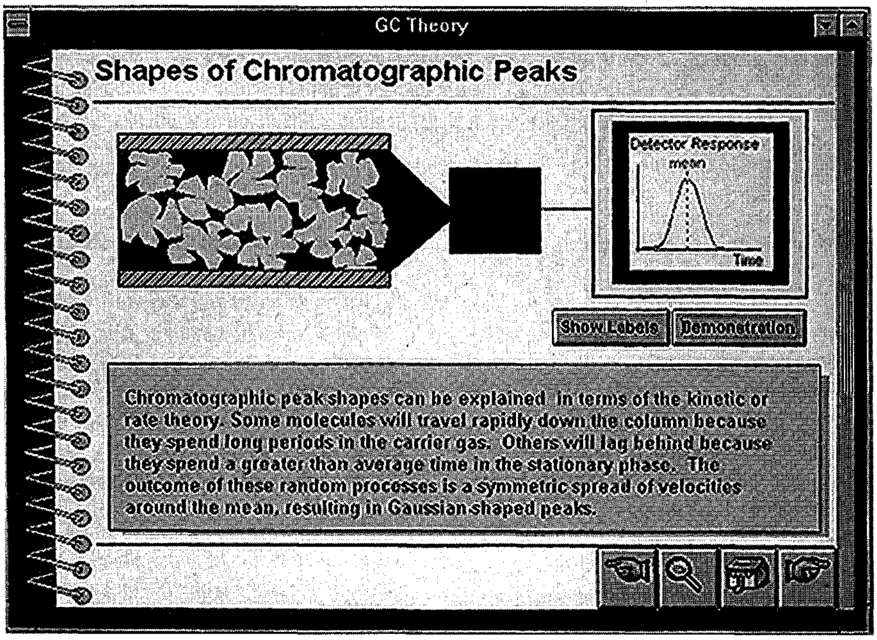

*The GC Softbook® from ATI Unicam provides training in gas chromatography, giving practical interactive learning of both instrumental and chemical aspects of the technique. The user can watch the animation demonstrating the working of an instrument; adjust parameters to see their effect; pursue their own lines of enquiry according to their needs, and return to areas of uncertainty. More information from ATI Unicam, York Street, Cambridge CB1 2PX, UK.*

### Gas analyser

The 610/74 High Purity Gas Analyser is a custom-designed system which routinely measures gases at low ppb levels. An information pack is available which contains a technical article, a comprehensive product report detailing the analyser, and several application notes. The notes describe the application of the 610/74 to the analysis of impurities in gases such as argon, helium, carbon dioxide, silane, boron trichloride and ethylene at levels down to less than 50 ppb.

*Copies of the pack from Paul Carter, ATI Unicam, York Street, Cambridge CB1 2PX, UK. Tel.: 01223 358866; fax: 01223 312764.*

### Moisture meter

The Shaw hygrometer gives unusual information regarding moisture in almost any manufactured or natural substance. It measures partial water vapour pressure rather than relative humidity which is of critical importance in any sample which may give trouble at a later date in storage. A rapid one second check on instant coffee, breakfast cereals, pills and tablets, is now possible. Moisture in dry liquids, such as transformer oil, LPG and fuel oil can also be quickly indicated.

With dry and wet alarms, a clear numeral LCD with a backlight, the Shaw hygrometer has a very wide range of measurement from less than one part per million of moisture to a dewpoint of plus 37°C (a range of one to 60 000). Discrimination is one part in two thousand and it has an interesting appearance during rapid reading changes of only one second from dry to wet. The 'Autodew' is supplied complete with detachable table stand, and a snap in facility for easy panel fitting. Although designed for moisture checks on any small sample, any increase in vapour pressure secures an alarm in still air over samples, or in flowing air or gas.

*Details from Shaw Moisture Meters, Westgate, Bradford BD1 3SQ, UK. Tel.: 01274 733582; fax: 01274 370151.*

### Multi-element analysis

The MDX1000 from Oxford Instruments is a low cost, fully integrated spectrometer. Using MDXRF—Multi-Dispersive X-Ray Fluoresence—high performance, simultaneous measurements can be made on both low to high atomic number elements.

The MDX1000 was designed with the industrial user in mind: it is robust and compact and can be located either in a laboratory or close to the process to be controlled. The spectrometer's detection system allows simultaneous measurements to be made over a wide concentration range, from high percentage to ppm levels in a variety of sample types.

Dedicated application packages and Oxford's *XpertEase* Windows™ XRF sofrware ensures ease of operation.

*Details from Oxford Instruments, Analytical Systems Division, 19/20 Nuffield Way, Abingdon, Oxon OX14 1TZ, UK. Tel.: 01235 532123; fax: 01235 535416.*

### Differential weighing

Many classical methods for the determination of the substance content of samples are based on the simple and clear differential weighing principle. The initial weights of a sample series are first determined by weighing and then a separation process, such as drying, centrifuging or sedimentation, separates the desired components. The residual weight is used to calculate the substance content. This procedure is exactly what the differential weighing software for the new AG, PG and SG balances from Mettler Toledo has been designed for.

The time required for calculations of routine applications in differential weighing is often considerable and in the absence of software support, manual management of the samples was prone to errors and the record keeping required by modern QA systems time consuming. Mettler Toledo's software manages, stores and calculates the data of up to 99 samples free from errors and automatically generates a clear, traceable record with raw data, results, sample numbers, time and date.

Sample management is more dependable because a bar code reader can be used. And it is possible to connect to a master lab information management system so that data can be sent to a computer in tabular form via the data interface of the balances.

*Details from Mettler-Toledo AG, CH-8606 Greifensee, Switzerland. Tel.: 1944 22 11; fax: 1 944 30 90.*

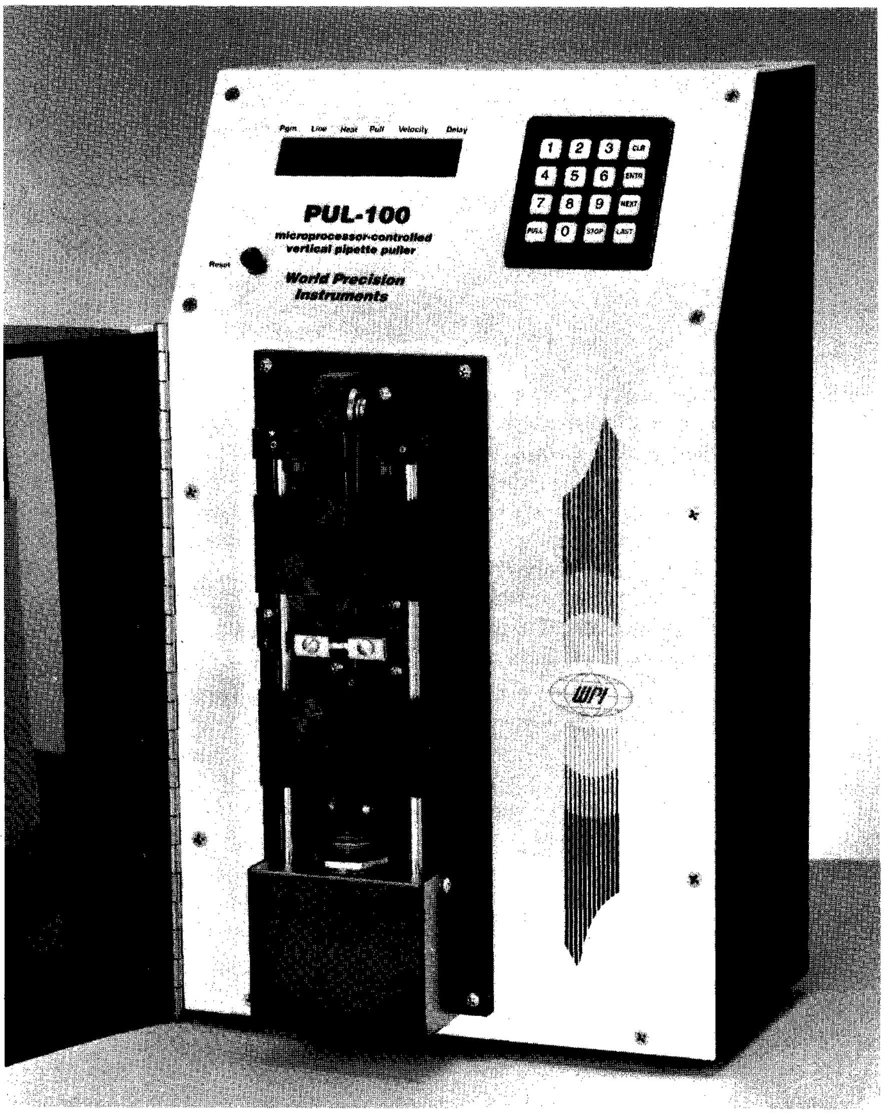

*The PUL-100 vertical micropipette puller will produce two virtually identical micropipettes with each pulling cycle. Ten programs can be stored in advance and tip diameter and taper can be varied for glass capillaries up to diameters of 2 mm. Precision ground stainless steel guide rails and capillary guides result in easy insertion and removal of glass capillary and finished micropipettes. The instrument contains at least two factory installed and tested programs: users can then modify or create their own programs for specific requirements. For more information contact World Precision Instruments, 175 Sarasota Center Boulevard, Sarasota, FL 34240-9258, USA. Tel.: 941 371 1003.*

### Balances

Mettler Toledo is offering a continuous range of compact and extremely rugged balances with built-in adjustment up to 32 kg. The models meet all of the demands of modern QA systems in plants—each AG and PG/SG precision balance has a built-in adjustment and test call-up (VariCal). VariCal ensures that users implement QA guidelines by informing them automatically as soon as the balance requires adjustment. At a keystroke, they can adjust or test their balances with the built-in adjustment weight or with selectable, external weights.

*Details from Mettler-Toledo AG (as above).*

### Chemistry information

The *ChemInform Electronic Journal* is now available on CD-ROM and includes the chemical abstracts journal *Fiz Chemie*. *ChemInForm* is equipped with a Windows front end and a retrieval system, specially adapted to the chemistry data contents. Chemical structure drawings are presented as facsimile pictures of the printed journal.

*For further information contact Fiz Chemie Berlin, Franklinstraße 11, 10587 Berlin, Germany. Tel.: 49 30 39977 111; fax: 49 30 39977 134.*

### Solartron

Solartron Instruments and Solartron Transducers have merged. The change is part of the companies' strategy to focus more closely on specific market sectors within the world-wide research, power generation, petrochemical and process industries. The new organization, which will be known as Solartron, is headed by John Thompson. Part of the Roxboro Group PLC, the new Solartron organization has three business units: data acquisition; laboratory analytical products; and transducers. Design, engineering, marketing and manufacturing functions are based at Farnborough, England, with further sales and service operations in Paris, Houston, Singapore and Beijing.

*For further information contact Pauline Taylor, Communications Department, Solartron, Victoria Road, Farnborough GU14 7PW, UK. Tel.: 01252 376666.*

### File Converters to support AIA data standard

GRAMS/386 includes file converters that support the AIA/netCDF FT-IR (v1.1.3), AIA/netCDF Mass Spectroscopy (v1.0.1b), and AIA/networkCDF Chromatograhpy (v1.0) Data Interchange Formats. The converters use version 2.3.2 of the netCDF software provided by Unidata Corp. These new converters join the expanding GRAMS/386 file conversion library which includes hundreds of file converters. The Analytical Instrument Association (AIA) has been working to define standards for technique-specific data interchange to meet the growing needs for data transfer and storage in the analytical laboratories today. Many of the major instrument vendors are members of the AIA and are contributing to its developments.

GRAMS/386 imports and processes data from hundreds of different analytical instruments—UV, UV-VIS, IR, FT-IR, NIR, NMR, LC, GC, HPLC, DAS and CE for example. GRAMS/386 includes a large processing library with routines such as peak fitting, baseline correction, data subtraction, smoothing, derivatives, peak picking, integration, and many more.

GRAMS/386 runs on 386, 486, on Pentium computers under Windows 3.1 or higher and Windows 95. A math co-processor and VGA display are required.

*For more information contact Christine Makis, Galactic Industries Corporation, 395 Main Street, Salem, NH 03079, USA. Tel.: 603 898 7600; fax: 603 898 6228. E mail: clm@galactic.com.*

### Performance enhancements to the Excalibur Plus System

Analytical requirements for the measurement of arsenic, selenium and antimony in a wide range of matrices are becoming increasingly demanding, especially for environmental legislation. The PS Analytical Excalibur Plus System has recently been improved to exceed the levels required to meet all legislation.

The instrumentation, which comprises the PSA 20.100 Random Access Autosampler, the PSA 10.004 Vapour Generator and the PSA 10.033 Excalibur Detector, couples the advantages of vapour generation to a unique PSA Atomic Fluorescence Detector. The new multi-reflectance filter assembly enables all of the hydride forming elements to be analysed simply by changing the boosted discharge hollow cathode lamp source. These improvements, coupled to increasing applications knowledge relating to the sample preparation and vapour techniques, have improved the detection limits by a significant amount. The detection levels achieved by the instrument in normal operating conditions are shown in the table.

Using the PSA software, which controls the operation of the instrument system and collects the data, it is possible to analyse samples for the elements over seven orders of magnitude, at analysis rates of up to 80 samples per hour. Recently, the range of analytes has been extended to include bismuth which is important in biological and metallurgical samples. The PS Analytical Excalibur provides significant economic and performance advantages over other expensive alternatives, for example, atomic absorption, inductively coupled optical emission and mass spectrometry.

*For further information contact PS Analytical Ltd, Arthur House, Unit 3, Crayfields Industrial Estate, Main Road, St Paul's Cray, Orpington, Kent BR5 3HP, UK. Tel.: 01689 891211; fax: 01689 896009.*

*Detection levels achieved by the Excalibur Plus (PS Analytical).*

| Analyte | Limit of detection ($3\sigma$) $\mu g\,l^{-1}$* |
|---|---|
| As | 0·01 |
| Se | 0·01 |
| Sb | 0·02 |
| Te | 0·02 |
| Bi | 0·05 |

*The limits of detection correspond to ten replicates on the blank measurements.

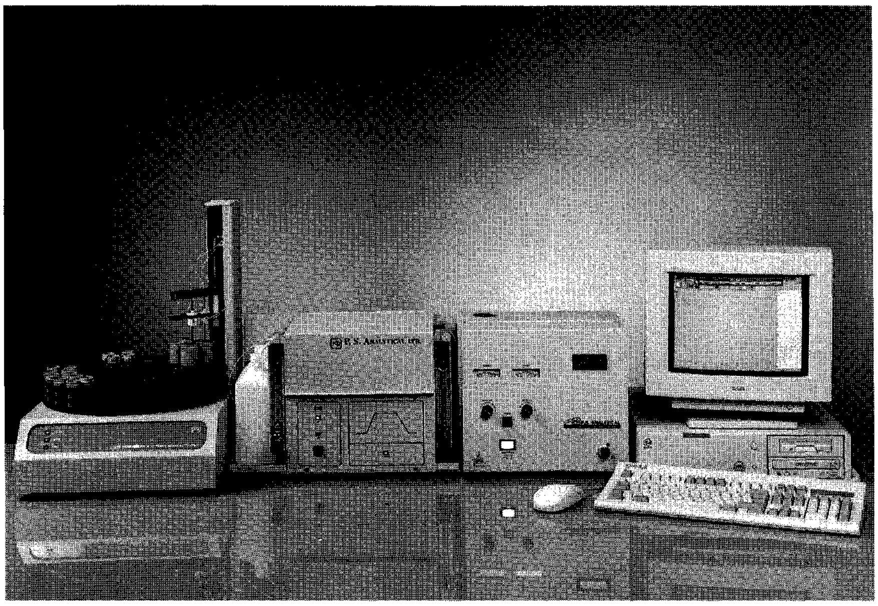

*The Excalibur Plus System from PS Analytical. The company's new laboratory facilities provide applications support so that the Excalibur Plus can be tailored to an individual laboratory's needs.*

### Environmental solutions

Hewlett-Packard S.A. has published its 1995/96 Environmental Solutions Catalog (Literature 5964-2045 LE). This includes information on many LC and GC methods, as well as troubleshooting, compound indexes, environmental literature from HP, column installation, helpful hints and environmental-methods guides.

Methods are separated into four sections: volatiles; semivolatiles; pesticides, herbicides and PCBs; and metals.

Each section has a matrix of related methods as well as helpful hints, and an LC and GC systems overview for working with the category of compounds presented. Within each section, new and updated methods are grouped by compounds and presented in a comprehensive format that includes method overview; chromatogram(s); recommended system; chromatographic parameters; chemical standards and test mixes; recommended columns and supplies; and references.

The troubleshooting section includes GC/MS and LC troubleshooting tips. A complete CAS index references all related methods and programs. The HPLC and GC environmental methods guides give information on sample prep, detection, columns recommendations, sample matrix and EPA reference methods for selected analytes.

*Enquiries to Hewlett-Packard, Enquiry Fulfilment Department, PO Box 533, 2130 AM Hoofddorp, The Netherlands.*

### Origin update

Microcal Software, Inc. has released Origin version 4.0. This release represents a substantial redesign of the software, including new data analysis and graphics tools, a user-friendly and powerful nonlinear curve fitter and a toolbar based user interface for instant access to analysis tools and common features. Origin 4.0 is compatible with Windows 95™ and is accompanied by extensive documentation.

Origin uses Windows to analyse, graph, and professionally present data for scientific and engineering applications. It requires minimal hardware (386/DX or higher, 4MR RAM, Windows 3.1 or later) and is very compact (4MB HD). A new 2 D graphics toolbar allows users to instantly access many graph types including new vector and polar graphs, as well as line, scatter, area, bar, pie, and statistical charts. New features in Origin 4.0, including baseline and peak analysis, designed for spectroscopy, improved Fast Fourier Transform (FFT) that facilitates digital signal processing, and a powerful nonlinear curve fitter with approximately 200 built-in functions, complement existing analytical tools such as smoothing, regression, and ANOVA. Toolbars are included to access the new features, simplifying the interface. Toolbars and menu commands can be customized or built using Origin's scripting language, LabTalk™, now a full featured C-like language.

*For a free evaluation kit contact Dr Santanu Bhattacharya, Microcal Software, Inc., One Roundhouse Plaza, Northampton, MA 01060, USA. Tel.: 413 586 2013; fax: 413 585 0126.*

